# Special Issue for “3D Printing of Drug Formulations”

**DOI:** 10.3390/ph16101372

**Published:** 2023-09-28

**Authors:** Touraj Ehtezazi

**Affiliations:** School of Pharmacy and Biomolecar Sciences, Centre for Natural Product Discovery, Liverpool John Moores University, Byrom Street, Liverpool L3 3AF, UK; t.ehtezazi@ljmu.ac.uk

Three-dimensional printing (3DP) is rapidly innovating the manufacturing process and provides opportunities that have never been seen before. Pharmaceutical dosage forms are no exception, and 3DP has been extensively investigated by researchers to realise this potential [1,2,3,4,5]. This Special Issue, entitled “3D Printing of Drug Formulations”, aims to showcase the latest developments and provide an overview of the diversity and complexity of 3DP of pharmaceutical dosage forms. The published articles in this Special Issue fill the gaps in our knowledge in achieving better medicines for patients. This Special Issue covers a wide range of dosage forms, including PLA filaments for bone generation, 3D-printed fast-dissolving oral films containing micro-ribbons and 3D-printed mucoadhesive gastroretentive hydrophilic matrices. Excitingly, this Special Issue presents two interesting review articles in the field of 3DP [6] and 4D printing [7].

To unfold this Special Issue, Khizer et al. (2023) employed fused deposition modelling (FDM) 3DP to manufacture floating tablets containing gabapentin for the treatment of overactive bladder. Their in vivo studies clearly demonstrated the extended-release nature of the 3D-printed formulations following oral administration to white albino rabbits [8]. 

The article by Akram Ghumman et al. (2023) is not directly related to 3DP; however, it is included in this Special Issue because the investigation achieved a disintegration time of 9.5 s for orodispersible tablets, which may be considered a target for fast-dispersing tablets/films prepared by FDM 3DP, as well as because of the methodology used for optimisation of the disintegration time [9]. In this study, FDM 3D-printed orodispersible tablets contained side channels to facilitate the disintegration of these tablets, a feature that cannot be incorporated via conventional tableting techniques. However, the disintegration time increased due to the thickness of the printing nozzle and the narrow diameter of the channels. This suggests the need to improve the printing resolution or further optimisation of the formulation. The disintegration time of paracetamol orodispersible tablets reduced to 2 min and 22 s and met the European Pharmacopeia (Ph. Eur) criteria when mannitol was included into the formulation at 10% *w/w*. Interestingly, Raman spectroscopy showed the presence of crystalline or amorphous paracetamol within the printed tablets [10]. 

The work by Pillai et al. (2023) demonstrated the use of FDM 3DP to generate personalised scaffolds containing copper nanoparticles to promote bone regeneration. The investigators clearly demonstrated the adhesion of human mesenchymal stem cells to the scaffolds with significant cell growth [11]. In the same field, Mohammad et al. (2023) employed FDM 3DP to produce scaffolds for improving bone generation through bone cell oxygenation and resistance to bacterial infections [12]. The scaffolds contained calcium peroxide and released oxygen with the help of native catalase in the recipient tissues.

In another study, FDM 3DP was employed to generate customised multi-compartmental capsules that could be used for high-throughput synthesis with the ability to recover key catalysts [13]. This will be important for the pharmaceutical industry to reduce the cost of the drug development process for identifying hit or lead constructs. Interestingly, mesh films were produced via FDM 3DP to maintain catalyst particles within a compartment. Certainly, this approach could have an application in the development of pharmaceutical dosage forms. Algellay et al. (2023) examined the use of micro-ribbons in FDM 3D-printed fast-dissolving oral films to improve the mechanical strength and disintegration time of printed films while using low-melting-temperature polymers. They found that only hydrophilic micro-ribbons of chitosan were able to improve the disintegration time of the films at low concentrations, perhaps by creating a network of hydrophilic channels within the films [14]. FDM 3D-printed oral films were also investigated by Lee et al. (2022) [15]. These investigators compared 3D-printed films of hydroxypropyl cellulose containing aripiprazole with solvent-cast counterpart films. They found that 3D-printed films disintegrated within 45 ± 4 s, while solvent-cast films dispersed within 63 ± 10 s. This is another great achievement for FDM 3D-printed films. The observed short disintegration time for 3D-printed films could be due to the higher surface roughness compared to solvent-cast films, allowing more exposure of the 3D-printed films to the disintegration media. Interestingly, PVA films disintegrated within 71 ± 12 s, which was shorter than the disintegration time for pure PVA films reported by Algellay et al. (2023). The difference could be due to the use of different disintegration techniques: Algellay et al. (2023) employed a tablet disintegration equipment, whereas Lee et al. (2022) used a Petri dish method. This highlights that a disintegration test method should be developed or accepted for evaluation of fast-dissolving oral films across the pharmaceutical community for consistent comparisons. 

The use of direct powder FDM printing provides the advantage of not requiring solvents to make filaments. In this regard, Malebari et al. (2022) employed direct powder extrusion 3D printing to produce personalised paediatric spherical minitablets of ritonavir or lopinavir with the diameter of 6 and 7 mm, respectively. Although the produced minitablets were not perfect spheres (as expected for FDM 3D printers for this size), the weight coefficient of variation was less than 8% for both formulations [16]. 
